# Sickness absence among mothers caring for a child with disability: Examining the impact of mechanical and psychosocial occupational exposures

**DOI:** 10.1016/j.ssmph.2024.101610

**Published:** 2024-01-28

**Authors:** Michael Yisfashewa Wondemu, Åsmund Hermansen, Pål Joranger, Idunn Brekke

**Affiliations:** a Norwegian Social Research, Section for Health and Welfare Research, Oslo Metropolitan University, Oslo, Norway; b Faculty of Social Sciences, Department of Social Work, Child Welfare and Social Policy, Oslo Metropolitan University, Oslo, Norway; c Faculty of Health Sciences, Department of Nursing and Health Promotion, Oslo Metropolitan University, Oslo, Norway; d Department of Childhood and Families, Norwegian Institute of Public Health, Oslo, Norway

**Keywords:** Children with disabilities, Sickness absence, Occupational mechanical exposures, Occupational psychosocial exposures

## Abstract

**Background:**

Sickness absence is more prevalent among mothers caring for children with disability compared to those caring for non-disabled children. Working in a poor working environment may worsen the impact of care burden on health outcomes among mothers of children with disabilities.

**Objective:**

The study investigated how sickness absences are associated with mechanical and psychosocial occupational exposures among mothers caring for children with and without disabilities.

**Methods:**

The study included children born between 2005 and 2013 and their respective mothers (*N = 147, 507*). Using register data from Statistics Norway, a Zero-Inflated Negative Binominal Regression was fitted to estimate the relationship between mechanical and psychosocial occupational exposures and sickness absence among employed mothers.

**Results:**

Mothers caring for children with disability had higher levels of sickness absences, even after adjusting for psychosocial and mechanical occupational exposures, and other possible confounding factors. When the occupational exposures analysed separately, both mechanical and psychosocial indices had a significant positive main effect on the number of sick days. The main effect of psychosocial exposure was no more significant in a simultaneous analysis, but mechanical exposure maintained its significant positive effect. However, we found no statistically significant differences in the number of sick absence days between mothers of children with and without disability based on their levels of psychosocial or mechanical job exposures.

**Conclusions:**

The findings emphasize the need of providing support to mothers caring for children with disability that help them manage occupational health risks.

## Highlights

• Sickness absence is more prevalent among mothers of children with disability.
• Mechanical and psychosocial job exposures increase sickness absences among mothers.
• No significant differences between mothers of children with and without disabilities based on job exposure levels.

## 1 Introduction

As more women have entered the labour market in the contemporary world, conflicts over how to best balance their work- and family responsibilities have grown (Campos-Serna et al., 2013; Kafetsios, 2007). Although that gender gap is narrowing, mothers still bear more responsibility than fathers for addressing the care-related demands of their families (Blair-Loy, 2009). The care burden responsibility appears to be stronger among mothers caring for children with disability than for those caring for non-disabled children (Brekke & Nadim, 2017; Burton et al., 2008; Morris & Zaidi, 2020). Studies conducted in the United States, Canada, Australia, Belgium and Norway, have demonstrated that, as a result of their increased caregiving responsibilities, mothers of children with disability reduce their work hours, earn less, and even withdraw from paid jobs (Bourke-Taylor et al., 2011; Brekke & Nadim, 2017; Burton et al., 2008; Hauge et al., 2013; Olsson & Hwang, 2006; van Dyck et al., 2004; Vinck & Brekke, 2020; Wondemu et al., 2022). Such demanding caregiving responsibilities may adversely impact parents health (Burton et al., 2008; Hauge et al., 2013), resulting in long term sickness absence (Brekke et al., 2017, Brekke et al., 2017; Brekke & Nadim, 2017; Wendelborg & Tøssebro, 2016).

Mothers are generally more susceptible to sickness absences than other women (Mastekaasa, 2000), and mothers of children with disability have higher sickness absence rates than mothers who do not. For example, using longitudinal register data from Norway, Brekke and Nadim (2017) found that compared to mothers of children without disability, long-term sickness absence among mothers caring for a child with chronic illness or disability were higher in the post-birth period. This is consistent with the findings of Brekke, Früh, et al. (2017) and Hauge et al. (2015). Hauge et al. (2015) reported that mothers of children with moderate to severe care needs were more likely to be on long-term sick leave than other mothers due to psychosocial problems. A Swedish study by Hedov et al. (2006), by contrast, reported no differences between parents of children with Down syndrome and those with non-disabling conditions.

Sickness absence among mothers of children with disability may be affected by social determinants of health, such as work and its associated conditions. Work is generally important for generating income and strengthening physical and mental health (Snashall & Patel, 2012). However, some types of work characterised by poor working conditions can be hazardous and constitute a health risk (Burgard & Lin, 2013; Snashall & Patel, 2012; van der Noordt et al., 2014; Waddell & Burton, 2006). Research has indicated that women have more work-related health problems than men, occupy fewer managerial positions, and enjoy less autonomy at work (Graveling, 2011; Schütte et al., 2015). Because they already have existing high care obligations, working in a poor environment may have a stronger adverse effect on the physical and mental health of mothers of children with disability, resulting in long-term sickness absence. To the best of our knowledge, the health effect of exposure to poor working conditions among mothers of children with disability has rarely been studied. Specifically, there are hardly any studies that have examined for the impact of mechanical and psychosocial job exposures among this group of mothers. Using register data from Statistics Norway, this paper investigated how sickness absences are associated with mechanical and psychosocial occupational exposures among mothers of children with and without disabilities. More knowledge on the adverse impact of occupational exposures on sickness absence may help in addressing work-related health burden of mothers of children with disabilities.

### 1.1 The health effects of occupational exposures

As a response to Marx’s (1992) recognition that contemporary labour is exploitative, a number of studies in the health sciences have focused on adverse occupational exposures that can impact health (Burgard & Lin, 2013). Several scholars, such as Butterworth et al. (2011); Fletcher et al. (2011); Hansen et al. (2015); Kim and von Dem Knesebeck (2015) report that workplace exposures, like physical and psychosocial hazards have a detrimental influence on physical and mental health. Despite contradictory results on differences in occupational exposures between men and women (Campos-Serna et al., 2013), research examining gender perspective on occupational health (e.g. Vermeulen & Mustard, 2000) has demonstrated that employed women face poorer working conditions than men, resulting in a greater health burden. According to a systematic review on gender inequalities in occupational health conducted by Campos-Serna et al. (2013), women appear to be affected differently than men by health problems due to occupational exposures. They tend to report poorer physical and mental health compared to men. For example, musculoskeletal symptoms were more commonly reported by women than men (Alterman et al., 2008; de Zwart et al., 2001; Emslie et al., 1999). This may be because they often engage in jobs that require repetitive hand movements and prolonged standing (Artazcoz et al., 2007).

Occupational exposures include psychosocial, mechanical (specific form of physical), biological, and chemical exposures. Previous studies have extensively investigated chemical, biological, and other forms of physical hazards. In this paper, we will focus on mechanical and psychosocial exposures. Mechanical job exposures include heavy lifting and physical labour associated with musculoskeletal disorder (Hermansen & Dahl, 2022), while psychosocial job exposures may be best exemplified by the aspects of Karasek’s (1979) model: psychological demands, decision latitude, and social support. Female-dominated occupations, such as shop salespersons and other sales personnel, nurses, nursing and care assistant, cleaners, and waitresses, are examples of professions that fall under both high mechanical and psychosocial exposures.

Studies document the relationship between mechanical job exposures and poor health outcomes among the working population (e.g. Harkness et al., 2003; Hoogendoorn et al., 1999; Kuiper et al., 1999; Sterud & Tynes, 2013). Mechanical occupational exposures, such as uncomfortable work positions, heavy loads, and repetitive movements commonly result in musculoskeletal pain. A three-year follow-up study of the general working population in Norway by Sterud and Tynes (2013) demonstrated a significant association between work-related mechanical exposures (e.g., highly demanding jobs, prolonged standing, and awkward lifting) and lower back pain. The results are comparable to what Harkness et al. (2003) found in a prospective cohort study of newly hired young workers: that mechanical exposures like lifting and pulling heavy weights were strong predictors of lower back pain. Occupational mechanical exposures have also been linked to an increase in sickness absences (Andersen et al., 2012; Hermansen & Dahl, 2022; Labriola et al., 2006; Sommer et al., 2016). For example, a longitudinal study conducted in Denmark by Sommer et al. (2016) found a statistically significant positive association between mechanical exposure and sickness absence. Using data from the Danish Work Environment Cohort Study, Labriola et al.’s (2006) study revealed that work-related mechanical exposures, such as working with arms lifted, bending over frequently, and repeated monotonous work, were associated with sickness absence. Their findings are consistent with a study by Hermansen and Dahl (2022) which validated an occupational mechanical job exposure index using data from nationwide Norwegian surveys and registry data. Their mechanical occupational index predicted a considerably increased risk of experiencing long-term sickness absences for women, based on both self-reported surveys and measured data from the national registry.

Burgard and Lin (2013) reported that psychosocial occupational exposures are linked to health risks that appear to progressively contribute to diseases, such as psychiatric illness, musculoskeletal pain, insomnia, and coronary heart disease. Using national survey data from Sweden and the United States, Karasek (1979) developed and validated the job strain model, which postulates that mental strain arises when job-related demands are high but control over job-related decision-making is low. According to the model, addressing health risks requires balancing and controlling the demands of work (e.g., work pace and hours). Karasek’s findings show that the interaction between heavy job-related demands and low latitude over job-related decision-making is associated with mental strain. Roughly a decade later, Karasek and Theorell (1990) added that psychosocial working conditions characterised by low autonomy, time pressure, and heavy job-related demands substantially affect physical and mental health. More recently, a prospective study in the United States by Fletcher et al. (2011) examined how physical demands on the job and the working environment affect people’s health. They found that individuals with long exposure to stressful situations at work have poor health, which accords with the results of Warren et al. (2004), who used data from a longitudinal study in Wisconsin to report that psychosocially demanding jobs are associated with poor health outcomes. Using both self-reported surveys and registry data, Le et al.’s (2023) study in Norway also found an association between psychosocial job exposure and long-term sick leave among both males and females.

Following previous research demonstrating the interplay between mechanical and psychosocial occupational exposures and sickness absence, we sought to investigate whether this association differs between mothers of children with and without disabilities. We expected that (1) mothers of children with disability would have higher levels of sickness absence than mothers of non-disabled children, after adjusting for psychosocial and mechanical exposures, and other covariates, and (2) the relationship between mechanical and psychosocial occupational exposures and sickness absence is stronger among mothers of children with disability.

## 2 Data and method

Data were obtained from the Statistics Norway’s (SSB) National Education Database, the Central Population Register, and the Event History Database (FD-Trygd). The data includes annual information on demography, education, employment and sickness absence for all individuals in Norway. Using FD-Trygd, children with disability were identified based on information about attendance benefits. The Norwegian Labour and Welfare Administration (NAV) provides attendance benefits to children with special needs based on their care needs which must last for at least two to three years, regardless of their parents' income. The sample consisted of mothers who were employed (*N = 147, 507*), and each paired with their first-born child. The sample was limited to children who were born between 2005 and 2013 due to differences in data structure between the birth cohort and job exposure indices.

### 2.1 Outcome measure

The outcome measure of this study was the number of sick days, a duration measure of sickness absence ranging from 0 to 365 days among mothers measured when their child was five years old. We reran the analysis of sickness absence two years after birth and found comparable results (results available upon request). Information on sickness absences was taken from FD-trygd, which only documents all physician-diagnosed absences lasting ≥16 calendar days, thus excluding any short-term spells of sick absences. Child disability status was measured by a dummy variable with a value of one if the child is disabled and zero if the child is non-disabled (control group). Children receiving attendance benefits (rates 2–4) were classified as children with a disability. The control group was comprised of mothers who during the observation period did not have any children receiving the attendance benefit.

### 2.2 Confounders

The analyses of the study were adjusted for birth cohort, number of younger siblings, mothers age at birth, marital status, child gender, immigrant background, education level, and sickness absence two years prior to childbirth. *Mothers age at birth* was measured as number of years. *Marital status* was measured by a dummy variable distinguishing whether the mother was married or not. *Immigrant background* was measured using three categories, distinguishing native Norwegians, 1st generation immigrants and 2nd generation immigrants. *Educational level* was divided into three levels: compulsory education and low, upper secondary education and college and university.

### 2.3 Exposure variables

The exposure variables were Hermansen and Dahl’s (2022) validated Mechanical Job Exposure Index and Le et al.’s (2023) validated Job Strain Index. Inspired by Hanvold et al. (2019), these two gender-specific job exposure matrix indices were constructed for use in Norwegian registry-based studies with the aim of addressing a lack of information on working conditions in Norwegian register data (Hermansen & Dahl, 2022; Le et al., 2023). The indices were based on five Norwegian nationwide surveys of living conditions concerning work environment in 2006, 2009, 2013, 2016, and 2019. Hermansen and Dahl’s mechanical exposure index contains eight exposures: heavy lifting (>20 kg), hands above shoulder height, heavy physical work, neck flexion, squatting or kneeling, forward bending, awkward lifting, and standing or walking. Each exposure was measured using different single-item and follow up questions (e.g., “Are you required to lift something weighing more than 20 kg on a daily basis?”, “Do you work with your hands raised above shoulder height?” and “Do you work in forward-leaning positions without using your hands or arms as support?”). The response options for the measures were dichotomized as exposed or not exposed. The Job Strain (Psychosocial) Index developed by Le et al. (2023) was based on Karasek’s Demand-Control Model and combines job demands with job control. Four items were used to measure job demand: quantitative demands, conflicting ways of doing things, insufficient resources, and contradictory requests. Six items were used to estimate job control or decision-latitude: deciding how to proceed with the work, deciding the tempo of work, making important decisions, applying skills, developing skills, and performing monotonous work. The Job Strain Index measures, like those for the Mechanical Job Exposure Index, had response options that were dichotomized as exposed or not exposed. Both indices were continuous variables based on the average dichotomized individual score in each Group-based Exposure Estimation or Job-Exposure Matrix (JEM) groups.

The mechanical and psychosocial exposure indices were constructed as the proportion of individuals within occupational groups that are exposed to the specific exposures (JEM scores). The mechanical index measures the mean proportion of mechanical exposures within each occupation and theoretically ranges from 0 to 100 %. The value 0 implies that no one has reported being exposed as part of a given occupation, whereas a value of 100 implies that everyone with a given occupational code has reported being exposed to all eight mechanical exposures. The job strain index also theoretically ranges from 0 to 100 percent, where higher values on the index represent higher degrees of demand and lower degrees of control. For a more detailed description of the mechanical index, see Hermansen and Dahl (2022) and for the job strain index, see Le et al. (2023). Our registry data included four-digit Norwegian standard STYRK-98 occupational codes for each employed mother; these codes were matched with the validated JEM scores. The aim was to identify the mothers psychosocial and mechanical occupational exposures based on their job titles. This was fulfilled by using their occupational codes at the time of childbirth and five years post-birth. For the small proportion of mothers who changed occupations during the 5 years after childbirth, their psychosocial and mechanical occupational exposures scores remained consistent.

### 2.4 Statistical method

To account for the excess of zero sick days and overdispersion, we used a Zero-Inflated Negative Binomial Regression (ZINB). Poisson regression is commonly used for analysing count data but its ability to handle overdispersion and excess zeros is limited (Hilbe, 2014). The variance in the number of sick days in our sample was approximately twice larger than the mean in the two groups of mothers. We analysed a Negative Binomial Regression (NBRG) output to test overdispersion, and found that alpha significantly differs from zero, suggesting a negative binomial model over Poisson. The distribution of number of sick days in the sample showed that 77 % of employed mothers had a value of zero, indicating that the distribution might be characterized by an inflated zero. Because our data only have information on long-term sickness absences, mothers with short-term spells appeared to have no sickness absences. The presence of excess zeros was tested by comparing the Akaike and Bayesian information criteria between ZINB and NBRG models (Hilbe, 2014). ZINB provided the best fit after this comparison. ZINB consists of two components: count and excess zero (logistic). The count component reported the duration of sick absence days, while the logistic component showed the probability of taking no long-term sick absence days. The logistic component was adjusted for child disability status, marital status, education level, and mechanical and psychosocial exposures indices. The marginal effects with robust standard errors of the ZINB were reported to examine the differences in the number of sick absence days between mothers of children with and without disabilities. We analysed both separate and combined impact of mechanical and psychosocial job exposures on sickness absence. In the separate analysis, we built two models, with the first testing for psychosocial job exposures and the second testing for mechanical job exposures. In the simultaneous analysis, we included both exposures in the same model. The simultaneous model was expanded by adding an interaction term between child disability status and psychosocial exposures and an interaction term between child disability status and mechanical exposures. We reran the interaction analysis in the separate models and found similar results (results available upon request). We reported the beta coefficients of predictors with robust standard errors in the interaction analysis. Marginal plots were provided to visually present the interaction effects. To test for the existence of a healthy worker effect, we conducted an additional analysis of the differences in the return-to-work following childbirth between the two groups of mothers using a Linear Probability Model (LPM).

All statistical analysis was performed using STATA® 16 with a significance level of *p* < 0.05. The sample characteristics of the two groups of mothers were compared using independent samples t-tests and Chi-square test.

## 3 Results

Table 1 presents descriptive statistics that describe sample characteristics measured at 5 years after childbirth. The sample included 143,309 mothers of children with disabilities and 4198 mothers of children without disabilities. Mothers caring for children with disability gave birth at slightly younger age (27.7) on average than among those caring for non-disabled children (28.4). Both group of mothers had higher proportion of male children than female: 67 % vs. 33 % for mothers of children with disability and 51 % vs. 49 % for mothers of children without disabilities. A higher proportion of the group of mothers of children without disability (45.6 %) were married than among the group of mothers of children with disability (43.5 %). There were differences in educational attainment between the two groups of mothers, with a higher proportion of college and university attainment among mothers of children without disabilities (56.2 % vs. 45.7 %). The descriptive table also showed that a higher proportion of mothers caring for a disabled child were native Norwegians (91.6 % vs. 90.2 %). Mothers of children with disability were absent from work for an average of 21 additional days more (48 days) than mothers of children without disability (27 days). Mothers caring for a child with disability had a higher long term sick days two years prior to childbirth than mothers caring for a non-disabled child. There was a slight but significant difference in the mean score of occupational mechanical and psychosocial exposure between the two group of mothers. Specifically, mothers of children with disability had a higher mean score on the mechanical exposure index (0.207) than mothers of children without disability (0.185). The mean score for psychosocial exposure was 0.338 for mothers caring for children with disability and 0.334 for those caring children without disabilities.

**Table 1:** Descriptive statistics of sample characteristics measured at 5 years after birth.

	Mothers of child with a disability *N = 4198*	Mothers of child without a disability *N = 143,309*	Signif. of difference
Mothers age at birth, yrs. (Mean, SD)	27.7 (5.3)	28.4 (5)	***
Child gender (%)			***
Male	67	51	
Female	33	49	
Number of days sickness absence, (Mean, SD)	47.7 (82.65)	27 (59.64)	***
Mechanical index, (Mean, SD, and range)	0.207 (0.116) (0.142–0.495)	0.185 (0.118) (0.005–0.505)	***
Psychosocial index, (Mean, SD, and range)	0.338 (0.053) (0.153–0.486)	0.334 (0.054) (0.143–0.486)	***
Immigrant background (%)			***
Native Norwegians	91.6	90.2	
1st generation	7.4	8.9	
2nd generation	0.9	0.8	
Educational level *(%)*			***
Compulsory education and less	19	14.3	
Upper secondary education	35.3	29.4	
College and university	45.7	56.2	
Marital status *(%)*			***
Married	43.5	45.6	
Unmarried	56.5	54.4	
Number of younger siblings *(%)*			
No younger siblings	17.9	16.9	***
1 and more siblings	82.1	83.1	
Sickness absence 2 years prior to childbirth (≥16-day spell) (*%)*	18	13	***

*N* = number of observations; The descriptive statistics for sickness absence 2 years prior to childbirth refers time point 2 years before childbirth.

Table 2, Table 3 present the results of ZINB analyses. We examined differences in the number of sick absence days between mothers of children with and without disabilities. The results indicate that mothers caring for a child with disabilities had about 19 additional days of sickness absence on average compared to mothers caring for children without disability, adjusting for occupational psychosocial and mechanical exposures and other confounding factors. When the occupational exposures analysed separately, both mechanical and psychosocial indices had a significant positive main effect on the number of sick days. This indicates that mothers with higher levels of mechanical or psychosocial exposures were more likely to have a higher sickness absence (Table 2). The main effect of psychosocial exposure was no more significant in the simultaneous analysis, but mechanical exposure continued to have its significant positive main effect (Table 3).

**Table 2:** Differences in the number of sick absence days between employed mothers of children with and without disabilities, adjusting for psychosocial and mechanical exposures separately.

Parameters	Coef.	Std. Err.	Coef.	Std. Err.
Count component				
Child disability (ref: non-disabled)	19.01***	1.245	18.74**	1.241
Psychosocial exposure index	17.17***	2.886		
Mechanical exposure index			21.59***	1.432
Excess Zero component				
Child disability status (ref: non-disabled) child)	−0.167***	0.004	−0.165***	0.007
Psychosocial exposure index	−0.266***	0.222		
Mechanical exposure index			−0.201***	0.111
*N*	147,507			147,507

Marginal effects from Zero-inflated Negative Binominal regression; ***p < 0.001 **p < 0.01; The count component was controlled for birth cohort 2005–2013, number of younger siblings, mothers age at birth, child gender, immigrant background, educational level, marital status, and sickness absence 2 years before birth; The excess zero component was adjusted for child disability, marital status, education level, and mechanical and psychosocial exposures indices; *N* = number of observations.

**Table 3:** Differences in the number of sick absence days between employed mothers of children with and without disabilities, adjusting for psychosocial and mechanical exposures simultaneously.

Parameters	Coef.	Std. Err.
Count component		
Child disability (ref: non-disabled child)	18.43***	1.237
Psychosocial exposure index	4.574	2.98
Mechanical exposure index	20.79***	1.499
Excess Zero component		
Child disability (ref: non-disabled child)	−0.161***	0.002
Psychosocial exposure index	−0.11***	0.229
Mechanical exposure index	−0.184***	0.117
*N*	147,507	

Marginal effects from Zero-inflated Negative Binominal regression; ***p < 0.001; The count component was controlled for birth cohort 2005–2013, number of younger siblings, mothers age at birth, child gender, immigrant background, educational level, marital status, and sickness absence 2 years before birth; The excess zero component was adjusted for child disability, marital status, education level, and mechanical and psychosocial exposures indices; *N* = number of observations.

**Fig. 1:**
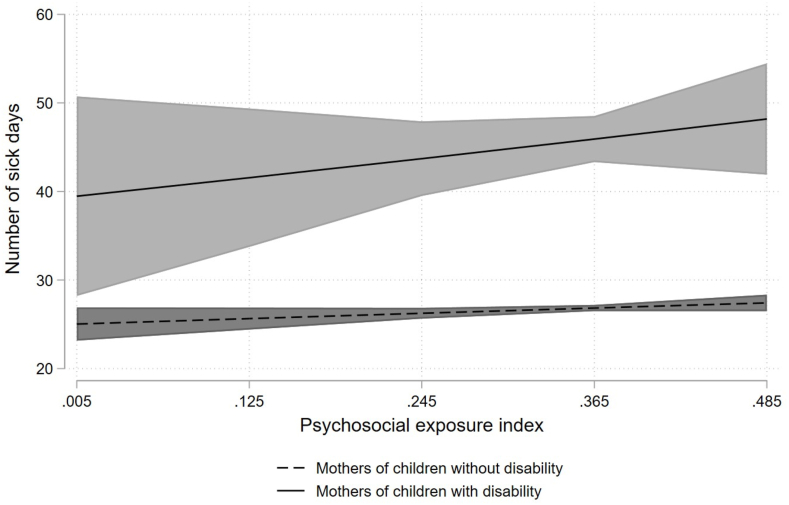
Predictive marginal effects of sickness absence by psychosocial exposure index and child disability status.

**Fig. 2:**
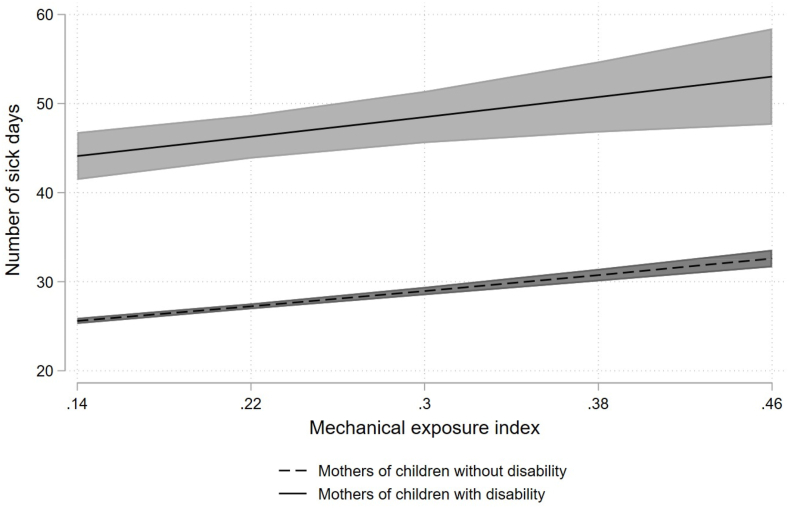
Predictive marginal effects of sickness absence by mechanical exposure index and child disability status.

Next, we examined if the relationship between mechanical and psychosocial exposures and sickness absence were stronger among mothers caring for children with a disability than for mothers caring for children with a disability. The results, which includes interaction terms between child disability status and occupational mechanical and psychosocial exposures, are presented in Fig. 1, Fig. 2 based on Table 4. Fig. 1 shows the predictive marginal effects of sickness absence (with 95 % Cls) by psychosocial exposure index and child disability status, while Fig. 2 shows comparable numbers for mechanical exposure index. The marginal plots lines in Fig. 1 shows a steeper slope for mothers caring for children with disability, but the ZINB analysis revealed no statistically significant interaction between child disability status and psychosocial exposure index (Table 4). Moreover, the parallel marginal plots lines in Fig. 2 indicate no interaction between child disability status and mechanical job exposures. This is substantiated by the absence of a statistically significant interaction between child disability status and mechanical exposure index (Table 4). The overall positive slope in Fig. 1, Fig. 2 implies that as mechanical and psychosocial job exposure increases, the number of sick absence days also increase for both groups of mothers. The figures also show that the level of sickness absence was higher among mothers caring for children with disability compared to those caring for children with a disability.

**Table 4:** The impact of child disability on the number of sick absence days of employed mother by psychosocial and mechanical occupational exposures.

Parameters	Coef.	Std. Err.	Exp (Coef.)	Coef.	Std. Err.	Exp (Coef.)
Count component						
Child disability status (ref: non-disabled)	−0.015	0.147	0.985	0.1*	0.0464	1.105*
Psychosocial exposure index	−0.207	0.0791	0.813	−0.194*	0.781	0.823*
Mechanical exposure index	0.158	0.393	1.171	0.16***	0.0387	1.173***
Child disability x psychosocial exposure index	0.318	0.426	1.374			
Child disability x mechanical exposure index				−0.0316	0.187	0.968
Constant	4.556***	0.0421	95.2***	4.468***	0.034	87.182***
Excess zero component						
Child disability status (ref: non-disabled)	−0.549***	0.205	0.577***	−0.715***	0.032	0.489***
Psychosocial exposure index	−0.518***	0.11	0.595***	−0.446***	0.111	0.64***
Mechanical exposure index	−0.887***	0.056	0.411***	−0.996***	0.0547	0.369***
Child disability x psychosocial exposure index	−0.463	0.599	0.629			
Child disability x mechanical exposure index				−0.912***	0.277	0.4***
Constant	1.265***	0.058	3.543***	1.155***	0.0591	3.174***
Alpha	−0.232***	0.006	0.792***	−0.232***	0.006	0.792***
N	147,507			147,507		

Zero-inflated Negative Binominal regression estimates; ***p < 0.001; **p < 0.01; *p < 0.05; *Analysis 1*: The interaction of child disability with psychosocial exposure index; *Analysis 2*: The interaction of child disability with mechanical exposure index; The count component was controlled for birth cohort 2005–2013, number of younger siblings, mothers age at birth, child gender, immigrant background, educational level, marital status, and sickness absence 2 years before birth; The excess zero component was adjusted for child disability, marital status, education level, and mechanical and psychosocial exposures indices; *N* = number of observations. The exponentiated coefficients (Exp (Coef.)) in the count component report incidence rate ratios (IRR), odds ratios (OR) in the excess zero component.

The excess zero components in Table 4 show that ZINB significantly predicts the probability of not taking long term sick days among mothers of children with and without disability after control for child disability status, marital status, education level, and mechanical and psychosocial exposures indices. This indicates that the existence of structural zeros is high and a ZINB model can better account for such zeros. Additionally, the excess zero component demonstrates that having a child with disability, and high mechanical and psychosocial exposures separately reduce the likelihood of not taking long term sick leave. The interaction term of mechanical exposure index and child disability status was found to be significant and negative, suggesting that having a child with disability and high mechanical job exposure reduce the probability of not taking long term sickness absence. However, there was no statistically significant interaction between child disability status and psychosocial exposure index.

Appendix A: Table 1 presents the differences in the return-to-work following childbirth between mothers of children with and without disability. The results showed that mothers caring for children with disability were less likely to return to work one year after childbirth than mothers caring for non-disabled children. For both groups of mothers, higher levels of mechanical and psychosocial occupational exposures were associated with a lower likelihood of returning to the labour market.

## 4 Discussion

In this paper, we have demonstrated how sickness absences are associated with mechanical and psychosocial occupational exposures among mothers of children with and without disabilities. Consistent with previous studies, we expected that mothers of children with disability would have greater levels of sickness absence than mothers of non-disabled children after adjusting for mechanical and psychosocial exposures and other covariates. The results of the present study indicate that mothers of a child with disabilities appear to have higher sickness absences five years after the child’s birth than mothers caring for a child without disability. Considering the findings of our study and those of similar studies by Bailey Jr et al. (2007); Brekke, Fruh, et al. (2017); Brekke and Nadim (2017); Burton et al. (2008); Hauge et al. (2013); Marks et al. (2002), we have shown that even after adjusting for mechanical and psychosocial occupational exposures that likely affect maternal sickness absences, there are still differences in sickness absences between mothers of children with and without disabilities. One possible explanation is that the higher care burden responsibilities may result in health problems and thus sickness absences. Mothers of children with disability may have missed significant time from work and reduce income because they had to dedicate more time than to care for their children, putting a burden on their mental and physical well-being and resulting in long-term sickness absence (see Burton et al., 2008; Olsson & Hwang, 2006; Wendelborg & Tøssebro, 2016; Wondemu et al., 2022). In the second aim of the study, we postulated that the relationship between mechanical and psychosocial occupational exposures and sickness absence would be stronger among mothers of children with disability than among other mothers. Contrary to our expectations, the findings demonstrated no interaction effect between child disability status and both psychosocial and mechanical occupational exposures between the two group of mothers. This suggests that the number of sick absence days between mothers of children with and without disability did not vary depending on their level of psychosocial or mechanical job exposures. The unexpected result may be attributed to Norway’s promotion of an inclusive labour market, which includes robust employment protection (e.g., flexible working hours and generous parental leave) and the active participation of women in the labour market (Eriksen, 2003; Sørvoll, 2015, pp. 1–48). These measures may help mothers caring for children with disability to manage the combined strain of care responsibilities and job exposures. The existence of a healthy worker effect could also explain why psychosocial and mechanical occupational exposures did not influence the differences in sick absence days between the two groups of mothers. Healthy worker effect is the consistent trend of the working population to have better health than the general population (Chowdhury et al., 2017).

In the return-to-work analysis, we observed that mothers of children with disabilities were less likely to return to work one year following childbirth. Mothers who returned to work were possibly healthier and better able to manage the combined strain of care responsibilities and high occupational mechanical and psychosocial exposures that those who did not. We also observed that mothers facing high occupational exposures were less likely to return to work. This could have led to an underrepresentation of mothers with poorer health who were exposed to high mechanical and psychosocial job exposures in our sample. Additionally, the negative interaction between mechanical job exposures and child disability status on the probability of not taking long-term sick absence days could be related to the existence of a healthy worker effect. Because mothers of children with disabilities with high mechanical job exposures in our sample could be healthier and better cope their work-related strain and care demands, they might often experience short term instead long-term sickness absence compared to other mothers.

Our empirical evidence contributes to the occupational health literature and emphasize the need of providing support to mothers caring for children with disability that help them manage occupational health risks. While we did not observe stronger influence of high levels of mechanical and psychosocial exposures on sickness absence among mothers caring for children with disability, we did find that the independent effect of such occupational exposures was positive and statistically significant. The impact of psychosocial exposures was no more apparent in the simultaneous analysis suggesting that the two exposures may explain the differences of sickness absence in a similar way. Overall, the results suggest that addressing mechanical and psychosocial exposures in the workplace can help reduce the prevalence of sickness absence among mothers with children with disability who are highly exposed to such exposures.

The present study findings are methodologically sound due to our use of rich longitudinal data and reliance on validated indices to examine exposure to poor working environments. To the best of our knowledge, the present study is the first registry-based cohort analysis examining the importance of mechanical and psychosocial job exposures in the interplay between sickness absences and caring for a child, among mothers of children with disability who are underexplored in the existing literature. The use of ZINB regression analysis increased the robustness of our model by accounting for the high presence of structural zeros in our data. It also helped us to demonstrate that both high mechanical and psychosocial exposures reduce the probability of not taking long-term sick leave among mothers of children with disability. One limitation of our study is that the use of a proxy measure for disability (i.e., attendance benefits) raises concerns about the accuracy with which children with disability can be identified from registry data. However, studies such as Wendelborg and Tøssebro (2016) indicate that in their sample of families caring for a child with developmental disability, nearly all children received attendance benefits, which indicates that it is appropriate to use attendance benefits to identify mothers caring for children with special needs. The use of attendance benefits also restricted our study from comparing variations in the impact of occupational exposures in sickness absence between mothers of children with and without disabilities, based on the nature and type of their children’s disabling conditions. Another notable limitation is that we were unable to investigate the impacts of psychological and mechanical job exposures on short-term sick absences. This is because the first 16 days of sick leave in Norway, which employers must pay for the employed mothers, were not documented in the registry data. Additionally, our registry data did not have information to account for the impact of living arrangements on childcare beyond the binary measure of marital status, such as the presence of significant other assisting with care.

## 5 Conclusion

This study has provided important insights into the impact of mechanical and psychosocial occupational exposures on sickness absence among mothers of children with and without disabilities. It contributes to the growing body of literature by demonstrating that mothers of children with disability had higher levels of sickness absences, even after adjusting for psychosocial and mechanical occupational exposures, and other possible confounding factors. When analysed separately, both high mechanical and psychosocial job exposures were associated with higher levels of sick absence days among employed mothers. However, no significant differences were found in the duration of sickness absence between the two groups of mothers relative to their levels of psychosocial or mechanical job exposures. These results underscore the need for future studies to investigate the long-term consequences of occupational exposures on sickness absence among mothers of children with disability. This is important because returned-to-work mothers who are exposed to high mechanical and psychosocial exposures may not be capable of coping with the care responsibilities and high occupational exposures over the long term, leading to increases in sickness absences and even withdrawal from the labour market. It would also be worthwhile to investigate how job characteristics, such as flexible working hours and social support at work, may moderate the relationship between sickness absence and caring for a child with disability among mothers who are exposed to high job exposures. Additionally, future research may look at whether the association between psychosocial exposures and sickness absence among mothers is moderated by mechanical exposures.

## Funding

10.13039/501100004787The Research Council of Norway funded this research as part of the BUDGET project (grant no: 301666).

## Availability of data and materials

The data used in this study are available from the Statistic Norway, but they are not publicly accessible because they were used under license for this study. The data may, however, be accessible from the authors upon reasonable request and with permission of the Statistic Norway.

## Consent for publication

Not applicable.

## Ethical statement

The ethical and legal aspects of this study have been thoroughly evaluated. The study was approved by the Regional Committee for Medical Research Ethics in south-eastern Norway (116474). The data used has been collected by Statistics Norway. We received permission by the Norwegian Data Protection Authority to access all the databases and records without written consent from the study participants. All methods were carried out in accordance with relevant guidelines and regulations.

## CRediT authorship contribution statement

**Michael Yisfashewa Wondemu:** Writing – review & editing, Writing – original draft, Validation, Software, Methodology, Investigation, Formal analysis, Conceptualization. **Åsmund Hermansen:** Validation, Supervision, Resources, Methodology, Conceptualization. **Pål Joranger:** Validation, Supervision, Methodology. **Idunn Brekke:** Validation, Software, Project administration, Funding acquisition, Conceptualization.

## Declaration of competing interest

The authors declare that they have no competing interests.

## Data Availability

The authors do not have permission to share data.
