# Supplementary material for: Sickness absence among mothers caring for a child with disability: Examining the impact of mechanical and psychosocial occupational exposures
Source: SSM Popul Health. 2024 Jan 28;25:101610. doi: 10.1016/j.ssmph.2024.101610 (PMC10840332; doi:10.1016/j.ssmph.2024.101610)
Supplement: Multimedia component 1 [file mmc1.docx]

**Appendix A**

**Table 1:** Differences in the return-to-work following childbirth between mothers caring for children with and without disability.

| Parameters | Returnees to labour market | |
| --- | --- | --- |
|  | Coef. | Std. Err. |
| Child disability status (ref: non-disabled) | -0.042*** | 0.006 |
| Mechanical exposure index | -0.054*** | 0.008 |
| Psychosocial exposure index | -0.159*** | 0.016 |
| Constant | 0.783 | 0.009 |

Linear Probability Model estimating the likelihood of mothers returning to labour market following childbirth; ***p < 0.001; The model was controlled for birth cohort 2005–2013, number of younger siblings, mothers age at birth, child gender, immigrant background, educational level, and marital status.

**Appendix B**

**Table 2:** Differences in the number of sick absence days between employed mothers of children with and without disabilities, adjusting for psychosocial and mechanical exposures simultaneously (includes socio-demographic coefficients).

| Parameters | Coef. | Std. Err. |
| --- | --- | --- |
| Count component |  |  |
| Child disability (ref: non-disabled) | 18.43^***^ | 1.237 |
| Psychosocial exposure index | 4.574 | 2.98 |
| Mechanical exposure index | 20.79*** | 1.499 |
| Number of younger siblings | 3.898*** | 0.173 |
| Child gender | -0.246 | 0.31 |
| Mother's age at birth | -0.068 | 0.037 |
| Immigrant background (ref: native) |  |  |
| 1st generation | -1.667*** | 0.558 |
| 2nd generation | 6.959*** | 1.856 |
| Educational level (ref: compulsory) |  |  |
| Upper secondary | -1.058 * | 0.531 |
| College and university | -4.893*** | 0.534 |
| Marital status (Married) | 0.424 | 0.374 |
| Sickness absence 2 years before birth | 3.975*** | 0.316 |
| Excess Zero component |  |  |
| Child disability (ref: non-disabled child) | -0.161*** | 0.002 |
| Psychosocial exposure index | -0.11*** | 0.229 |
| Mechanical exposure index | -0.184*** | 0.117 |
| *N* | 147507 |  |

Marginal effects from Zero-inflated Negative Binominal regression; ***p < 0.001; The count component was adjusted for birth cohort 2005–2013; The excess zero component was adjusted for child disability, marital status, education level, and mechanical and psychosocial exposures indices; *N=* number of observations.
